# Characterizing immune evasion in FFPE tissue sections - a new method for measuring cellular interactions via multiplexed phenotype mapping and spatial point patterns

**DOI:** 10.1186/2051-1426-2-S3-P259

**Published:** 2014-11-06

**Authors:** Clifford Hoyt, Chichung Wang, Kristin Roman, Kent Johnson, Peter Miller, Elizabeth Mittendorf

**Affiliations:** 1 Perkin Elmer, Inc., USA; 2 MD Anderson Cancer Center, University of Texas, Houston, TX, USA

## Background

Full realization of the potential of immunotherapy will require biomarkers that capture immuno-tumor interactions. This will most effectively be achieved by phenotyping cells in-situ in intact tissue sections. Such methods will potentially predict response to drugs and monitor efficacy and onset of resistance. Existing methods are limited. Here we report a novel integrated approach, utilizing highly multiplexed immunofluorescence labeling, imaging-based analysis to capture expression and cellular interactions in tumor and the microenvironment, and statistical analysis. Of particular interest is applicability of this approach to intact FFPE tissue sections, amenable to routine pathology workflow. The capability of this technology to show the range of immune-tumor interactions is demonstrated on a set of breast cancer samples.

## Methods

Breast specimens were labeled for CD4, CD8, CD20, PD-L1, Foxp3, cytokeratin, and DAPI with a new serial same-species fluorescence labeling approach utilizing tyramide signal amplification (Opal™), and microwave-based antigen retrieval and antibody stripping, which enables specific, non-interfering, balanced labeling. Samples were imaged on a multispectral slide analysis system, and analyzed with pattern recognition software, to segment tissue into tumor and stroma, and phenotype cells using a new multinomial logistic regression learning capability that is intuitive, fast, and reliable across clinical variability. Cells were phenotyped into categories of tumor, killer T, helper T, regulatory T, B cells, producing cell phenotype maps retaining spatial arrangements. Imagery was assessed by a pathologist for segmentation and cell phenotyping accuracy, and then evaluated using spatial point pattern analysis. As an example statistic, nearest-neighbor calculations are performed to determine percentage of tumor cells having killer T cells within 25 microns.

## Results

**Figure 1:**
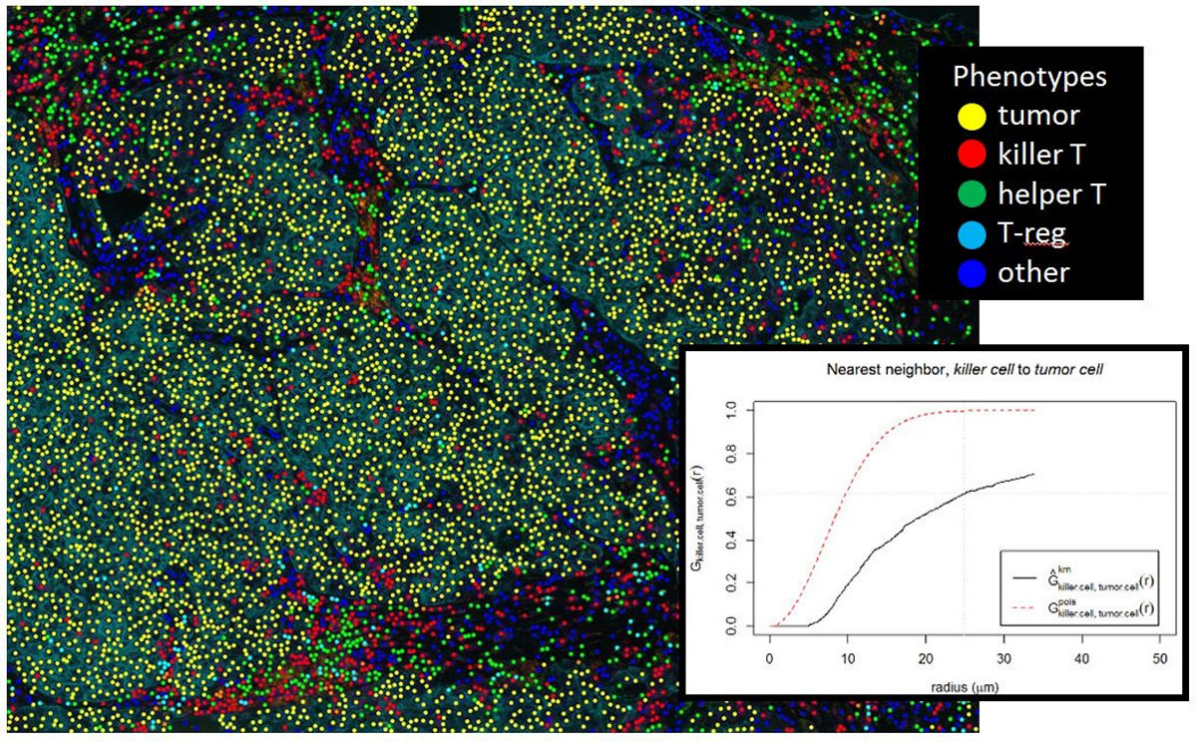


Labeling of five breast cancer samples demonstrated specificity and sensitivity estimated by a pathologist to be greater than 95% accurate. The five cases presented significantly different killer T and tumor cell interaction statistics. As an example, nearest-neighbor analysis indicates the five cases had, respectively, 54%, 10%, 61%, 33%, and 55% of tumor cells having killer T cells within 25 microns (Example case in Figure 1). This is one metric of many that will be presented, that clearly delineate immune-tumor interactions.

## Conclusions

The approach shows reliable detection of immune cell phenotypes and segmentation of tumor and stroma, to accurately assess interaction between immune and tumor cells. We believe results support the feasibility of a practical and viable clinical workflow, in which immune response assessment is automated by computer and results are reviewed by pathologists to assure data quality.

